# Patient Characteristics Following Surgery for Spinal Metastases: A Multicenter Retrospective Study

**DOI:** 10.1111/os.12551

**Published:** 2019-12-10

**Authors:** Li Yang, Feng Wang, Hao Zhang, Xiong‐gang Yang, Hao‐ran Zhang, Ji‐kai Li, Rui‐qi Qiao, Guo‐chuan Zhang, Yong‐cheng Hu

**Affiliations:** ^1^ Department of Bone Tumor Tianjin Hospital Tianjin China; ^2^ Graduate School Tianjin Medical University Tianjin China; ^3^ Department of Musculoskeletal Tumor Third Hospital of Hebei Medical University Hebei China

**Keywords:** Epidemiological study characteristics, Spinal metastases, Surgical treatment, Univariate analysis

## Abstract

**Objectives:**

To summarize the epidemiological characteristics of patients following surgery for spinal metastases retrospectively and make a univariate analysis to identify independent variables that could affect the operation decision making.

**Methods:**

This was a multicenter retrospective review of patients with spinal metastasis who were treated with surgery from 1 January 2007 to 31 July 2019. Basic clinical data were analyzed retrospectively by univariate analysis to identify independent variables that could affect the decision of operation modalities, including gender, age, spinal metastatic site, Frankel score, Karnofsky performance score (KPS), spinal instability neoplastic score (SINS), visual analogue scale (VAS), Tokuhashi score, urinary and fecal incontinence, spinal pathological fracture, primary tumor, extraspinal metastasis, visceral metastasis, and bone lesion (osteolytic, osteoblastic or mixed).

**Results:**

A total of 580 patients including 332 males and 248 females were enrolled in the study with an average age of 58.26 years old (range, 13–86 years old). The most common spinal metastatic level was the thoracic vertebra (190 [32.76%]), followed by the lumbar vertebra (146 [25.17%]), cervical vertebra (47 [8.10%]), and sacral vertebra (35 [6.03%]). Metastases involving more than two sites of the cervical, thoracic, lumbar, and sacral vertebrae arose in 162 (27.93%) patients. For primary tumor, there were 198 (34.14%) cases of lung cancer, 41 (7.07%) cases of kidney cancer, 39 (6.72%) cases of breast cancer, 38 (6.55%) cases of gastrointestinal cancer, 35 (6.03%) cases of lymphoma and myeloma, 25 (4.31%) cases of prostate cancer, 24 (4.14%) cases of liver cancer, 23 (3.97%) cases of mesenchymal tissue sarcoma, 20 (3.45%) cases of thyroid cancer, and 84 (14.48%) cases were tumor with unknown origin. Sixty‐three (10.86%) patients received minimally invasive surgery, 460 (79.31%) patients received palliative surgery, and the remaining 57 (9.83%) received tumor resection. According to the univariate analysis, the KPS score, SINS score, VAS score, Tokuhashi score, urinary and fecal incontinence, spinal pathological fracture, and bone lesion (osteolytic, osteoblastic or mixed) were independent and favorable factors affecting the surgery modalities.

**Conclusions:**

Surgical treatment for spinal metastases was mainly to relieve pain, rebuild spinal stability, improve nerve function, control local tumors, and improve the quality of life of patients. For middle‐aged and elderly patients with good general conditions, severe pain, spinal pathological fracture, spine instability and without urinary and fecal incontinence, early surgical treatment should be actively carried out.

## Introduction

Spinal metastases are the most common type of bone metastases with a prevalence of 30%–70% in cancer patients and 5%–10% metastases may be associated with epidural spinal cord compression (ESCC) leading to impaired mobility, neurologic deficits, and decreased quality of life^1^, ^2^, ^3^, ^4^.

In order to relieve pain, improve nerve function, control local tumors, and improve quality of life for patients, surgery is more and more widely performed, including minimally invasive surgery, palliative surgery, or radical surgery. In turn, the majority of studies report a significant clinical effect for carefully selected spinal metastatic patients^5^, ^6^, ^7^. Flavio Tancioni *et al*.^8^ reported 25 consecutive patients with a diagnosis of ESCC from solid primary tumors. These patients were treated with minimally invasive surgery, with 96% clinical remission of pain and 88% improvement of neurological deficit after 2 weeks. Masuda *et al*.^9^ assessed the surgical outcomes of 44 patients treated with posterior decompression and stabilization and reported that the Frankel score and Eastern Cooperative Oncology Group performance status (ECOG‐PS) improved in all patients after surgery. Boriani *et al*.^10^ also applied total en‐bloc spondylectomy for 165 patients, and reported that all patients had neurologic deficits improvement and the local recurrences recorded were just 15.28% after 25 years.

However, there still remains some problems when treating spinal metastasis with surgery. Complications must be considered after surgery, such as intra‐operation bleeding, spinal cord injury, and hematoma^11^, ^12^. Furthermore, the purposes for spinal metastasis treatment are usually different from visceral metastases, which makes the treatment concept, preoperative evaluation, and treatment strategy of spinal metastasis become irregular and arbitrary^13^, ^14^. At the same time as the rapid development of immuno‐therapy, endocrine therapy, radiotherapy, and chemotherapy (especially targeted therapy), a multidisciplinary combined therapy of spinal metastasis has become a trend^15^, ^16^, ^17^. Therefore, indications and contraindications for spinal metastasis surgery treatment should be clearly understood.

Accordingly, a multicenter retrospective study was performed with the aim of: (i) summarizing the epidemiological characteristics of patients following surgery for spinal metastases; (ii) making a subgroup analysis to identify independent and favorable factors which could affect the surgery selection; and (iii) helping clinicians make a more appropriate surgery decision for patients with spinal metastasis.

## Patients and Methods

### *Participants*

This was a multicenter retrospective review of patients with spinal metastasis who were treated with surgery from 1 January 2007 to 31 July 2019. All patients met the following inclusion criteria: (i) patients diagnosed with spinal metastasis precisely by clinical imaging examination (CT, MRI, ECT or PET‐CT) or pathological examination; (ii) patients with hematological malignancy spinal metastasis, including lymphoma and myeloma; (iii) patients who were treated by surgical intervention; and (iv) patients whose observation indicators below could be retrospectively analyzed.

Exclusion criteria for this review were: (i) patients with impaired spinal cord function due to other diseases, such as primary spinal tumors, spinal tuberculosis, or spinal degenerative diseases; (ii) outpatients; (iii) patients with another spinal surgery aside from the metastatic tumor; and (iv) patients undergoing biopsies as the only surgical intervention.

### *Operation Category*

Operations applied for patients were mainly divided into minimally invasive surgery and aggressive surgery based on the operation invasiveness.

Minimally invasive surgery was defined as techniques which had lower associated soft tissue damage and shorter hospital lengths of stay, including percutaneous vertebroplasty (PVP) or percutaneous kyphoplasty (PKP).

For aggressive surgery, palliative surgery was applied for the purpose of improving impaired mobility, neurologic function, and quality of life, but the tumor was not resected completely. Posterior laminectomy decompression and subtotal corpectomy (combing with vertebroplasty and microwave ablation or not) were included.

For the purpose of removing the tumor completely, radical surgery was performed for patients, including total or piecemeal vertebrectomy, piecemeal or total en‐bloc spondylectomy.

### *Observation Indicators*

Indicators were collected including gender, age, primary malignancy type, spinal metastatic level, spinal pathological fracture, urinary and fecal incontinence, extraspinal metastasis, visceral metastasis, bone lesion, Frankel score, Karnofsky performance score (KPS), visual analogue scale (VAS), spinal instability neoplastic score (SINS), and Tokuhashi score.

Primary malignancy type was defined as the origin of spinal metastatic tumor, such as lung cancer, breast cancer, and kidney cancer, among others.

Spinal metastatic level was defined as the location where the metastatic tumor existed. Based on the anatomical structure of the spine, it was divided into the cervical vertebra, thoracic vertebra, lumbar vertebra, sacral vertebra, and trans‐segmental metastasis.

Spinal pathological fracture was defined as vertebral body or appendix fractured due to the tumors based on examinations with X‐rays, CT, or MRI.

Extraspinal metastasis was defined as patients with bone metastasis other than that occurring in the spine (such as rib, femur, tibia, fibula).

Bone lesion was identified based on the function of osteoblasts and osteoclasts, including osteolytic, osteoblastic, and mixed, through examinations with X‐rays or CT.

Frankel score classification provided an assessment of spinal cord function, which was divided into five grades of A, B, C, D, and E based on the degree of spinal cord injury. Grade A meant complete neurological injury with no motor and sensory function, Grade B meant preserved sensation only, Grade C meant preserved nonfunctional motor, Grade D meant preserved functional motor, and Grade E meant normal motor and sensory function^18^.

KPS score was used to assess the functional status of patients. From 0 to 100, patients with no symptoms were scored at 100, patients who died were scored at 0. Generally speaking, KPS score above 80 was considered to be self‐care level, 50–70 was considered into half self‐care level, and 50 was considered patients needing help from others^19^.

VAS score was a measure of pain intensity and it was a continuous scale comprised of a horizontal (called horizontal visual analog scale) or vertical (called vertical visual analog scale) scale. For pain intensity, the scores could be from 0–10, which was determined by measuring the distance (mm) on the 10 cm line between the “no pain” anchor and the patient's mark^20^.

SINS score was applied for assessing the spinal instability. It contained the lesion location, mechanical pain, bone lesion, radiographic spinal alignment, vertebral body collapse, and posterolateral involvement. The total score was 18 (1–6 meant stable, 7–12 meant potentially stable, and 13–18 meant unstable)^21^. Tokuhashi score was a prognostic evaluation of patients which was based on KPS score, numbers of extraspinal metastasis, numbers of vertebra bodies, visceral metastasis, primary malignancy type, and spinal cord palsy. The score was from zero to 15, usually divided into 0–8 with overall survival less than 6 months, 9–11 with overall survival between 6 and 12 months, and 12–15 with overall survival more than 12 months^22^.

### *Statistical Analysis*

Measurement data (age, intra‐operation bleeding, and operation time) were expressed as their mean, with the minimum and maximum values compared with the *t*‐test. Counting data (gender, primary tumor, and neurological assessment etc.) were compared using the *χ2*‐test. All statistical analyses were performed using IBM SPSS Statistics 22.0, and a two‐tailed *P* < 0.05 was considered significant difference statistically.

## Results

### *Cohort Characteristics*

As shown in Table 1, a total of 332 male and 248 female patients were enrolled in the study with an average age of 58.26 years old (range, 13–86 years old), an average intra‐operation bleeding of 1334.98 mL (range, 5–9000 mL), and an average operation time of 216.31 min (range, 60–680 min).

**Table 1:** Characteristics of the studied cohort (n = 580)

	Lung cancer	Kidney cancer	Breast cancer	Prostate cancer	Thyroid cancer	Liver cancer	Colorectal cancer	Gastric cancer	Myeloma and lymphoma	Mesenchymal tissue sarcoma
**Gender**										
Male	120	35	0	25	5	20	10	15	19	9
Female	78	6	39	0	15	4	11	2	16	14
**Age (year)**										
≤44	24	3	10	0	0	2	1	1	2	8
45–59	79	13	18	4	10	9	11	6	11	6
60–74	89	21	10	15	8	13	8	8	22	9
75–89	6	4	1	6	2	0	1	2	0	0
**Spinal metastatic site**										
**Cervical vertebra**										
Single segment	14	5	0	2	1	4	0	0	0	1
Multiple segment	5	0	2	0	1	1	0	0	0	0
**Thoracic vertebra**										
Single segment	30	9	9	4	3	3	5	5	8	5
Multiple segment	25	8	7	7	4	4	3	3	5	5
**Lumbar vertebra**										
Single segment	39	6	5	2	6	3	6	2	9	6
Multiple segment	16	2	2	1	0	1	1	0	5	2
**Sacral vertebra**	8	1	0	3	1	3	3	1	0	3
**Trans‐segmental metastasis**	61	10	14	6	4	5	3	6	8	1
**Extraspinal metastasis**										
Yes	96	17	14	14	8	9	7	7	14	6
No	102	24	25	11	12	15	14	10	21	17
**Visceral metastasis**										
Yes	31	6	3	2	5	6	9	2	0	5
No	167	35	36	23	15	18	12	15	35	18
**Spinal pathological fracture**										
Yes	69	15	21	9	6	8	8	5	20	9
No	129	26	18	16	14	16	13	12	15	14
**Bone lesion**										
Osteolytic	67	15	11	8	5	7	4	8	24	9
Osteoblastic	5	0	0	1	0	0	0	0	0	0
Mixed	1	0	0	1	0	0	0	0	0	1
Unknown	125	26	28	15	15	17	17	9	11	13

	Unknown origin	Reproductive system tumors	Esophageal cancer	Bladder cancer	Pancreas cancer	Others	Totally (n)
**Gender**							
Male	48	2	7	3	5	9	332
Female	36	10	2	3	0	12	248
**Age (year)**							
≤44	8	2	0	1	1	7	70
45–59	27	7	2	2	2	10	217
60–74	38	3	7	3	2	3	259
75–89	11	0	0	0	0	1	34
**Spinal metastatic site**							
**Cervical vertebra**							
Single segment	4	0	1	0	0	1	33
Multiple segment	2	0	0	0	0	0	14
**Thoracic vertebra**							
Single segment	7	2	3	1	3	2	99
Multiple segment	14	0	1	1	1	6	91
**Lumbar vertebra**							
Single segment	16	3	1	2	0	5	111
Multiple segment	7	0	0	0	0	0	35
**Sacral vertebra**	8	2	0	1	0	3	35
**Trans‐segmental metastasis**	26	5	3	1	1	4	162
**Extraspinal metastasis**							
Yes	32	3	2	2	0	5	237
No	52	9	7	4	5	16	344
**Visceral Metastasis**							
Yes	11	0	2	0	2	6	90
No	73	12	7	6	3	15	490
**Spinal pathological fracture**							
Yes	29	3	3	2	2	3	212
No	55	9	6	4	3	18	368
**Bone lesion**							
Osteolytic	23	3	4	3	2	2	195
Osteoblastic	2	0	0	0	0	0	8
Mixed	0	0	0	0	0	0	3
Unknown	59	9	5	3	3	19	374

**Figure 1:**
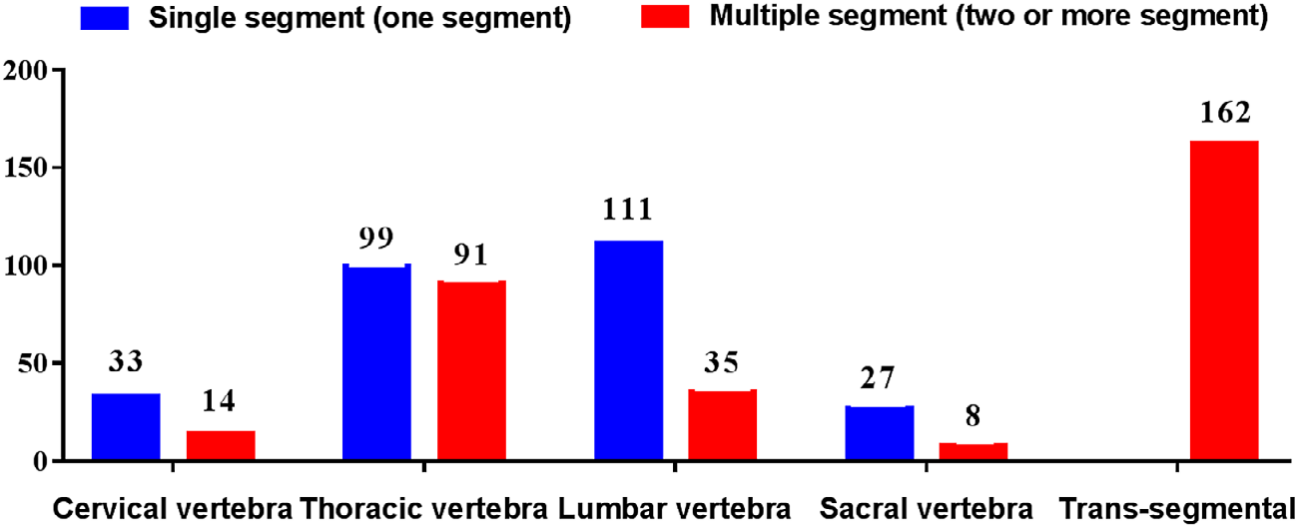
Spinal metastatic level among the 580 patients. The most common spinal metastatic site was the thoracic vertebra (190 [32.76%]), followed by the lumbar vertebra (146 [25.17%]), cervical vertebra (47 [8.10%]), sacral vertebra (35 [6.03%]) and trans‐segmental metastasis (162 [27.93%]). Only one single segment metastasis was presented in 247 (42.59%) and two or more segment metastasis was in 333 (57.41%).

The most common spinal metastatic level was the thoracic vertebra (190 [32.76%]), followed by the lumbar vertebra (146 [25.17%]), cervical vertebra (47 [8.10%]), and sacral vertebra (35 [6.03%]). Metastases involving more than two sites of the cervical, thoracic, lumbar, and sacral vertebrae arose in 162(27.93%) patients. Among these patients, only one single segment metastasis was presented in 270 (46.55%) patients and two or more segment metastases were presented in 310 (53.45%) patients (Fig. 1).

**Figure 2:**
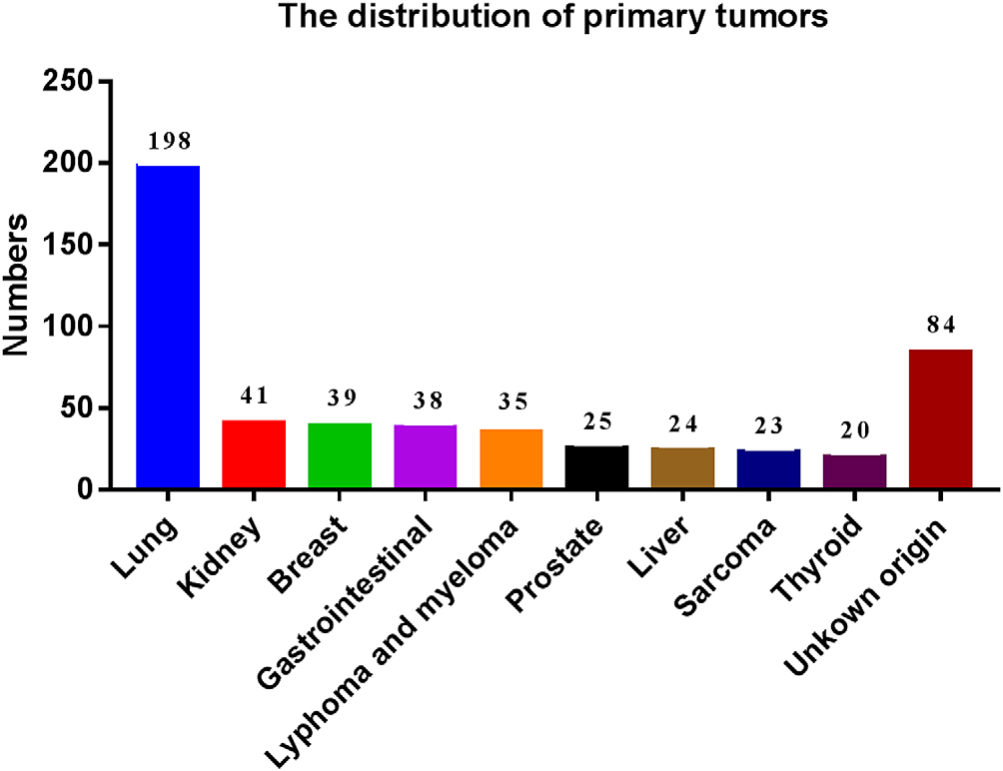
Distribution of the primary tumors in 580 patients with spinal metastasis treated with surgery. Lung cancer was the most one in 198(34.14%) cases. Kidney cancer, breast cancer, gastrointestinal cancer, lymphoma, and myeloma did not show significant difference. Prostate cancer, liver cancer and mesenchymal tissue sarcoma were nearly at the same. 84 (14.48%) cases were with unknown origin of tumor but with clear pathological examinations.

For primary tumors, there were 198 (34.14%) cases of lung cancer, 41 (7.07%) cases of kidney cancer, 39 (6.72%) cases of breast cancer, 38 (6.55%) cases of gastrointestinal cancer, 35 (6.03%) cases of lymphoma and myeloma, 25 (4.31%) cases of prostate cancer, 24 (4.14%) cases of liver cancer, 23 (3.97%) cases of mesenchymal tissue sarcoma, 20 (3.45%) cases of thyroid cancer, and 84 (14.48%) cases were with unknown origin of tumor (Fig. 2).

**Figure 3:**
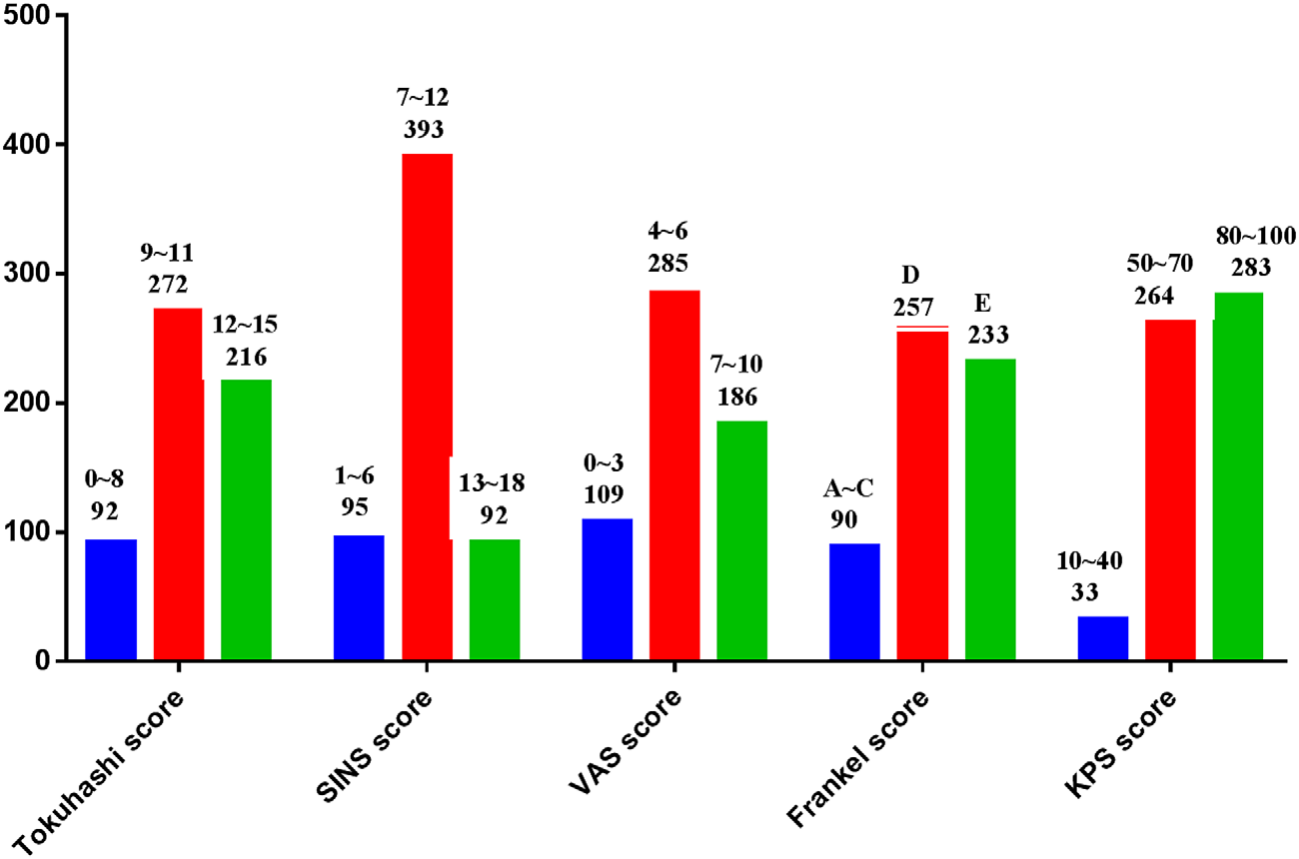
Distribution of Tokuhashi score, SINS score, VAS score, Frankel score and KPS score in 580 patients treated with surgery. Tokuhashi score more than nine was shown in 488 (84.14%) patients; 485 (83.62%) patients presented spinal instability with SINS score more than 7; 471 (81.21%) patients presented pain with VAS score more four. As for neurological impairment, 90 (15.52%) patients presented paralysis.

Four hundred and seventy one (81.21%) patients presented unbearable pain with an average VAS score of 7.12 (range, 0–9). As for neurological impairment, 90 (15.52%) patients presented paralysis including Frankel A in 27 patients, Frankel B in 13 and Frankel C in 50 patients. Furthermore, 485 (83.62%) patients presented spinal instability and the average SINS score of 8.02 (range, 7–18). More details were presented in Fig. 3.

### *Operation Category and Univariate Analysis*

In this cohort study, 63 (10.86%) patients received minimally invasive surgery (including 58 PVP and five PKP). Four hundred and sixty (79.31%) patients received palliative surgery (including 290 posterior laminectomy, 155 subtotal corpectomy, 15 subtotal corpectomy combined with microwave ablation and vertebroplasty) and 57 (9.83%) patients received radical surgery (including 36 total vertebrectomy and 21 total en‐bloc spondylectomy). The results of univariate analysis were shown in Table 2, with KPS score, SINS score, VAS score, Tokuhashi score, urinary and fecal incontinence, spinal pathological fracture, and bone lesion (osteolytic, osteoblastic or mixed) being independent and favorable factors affecting the surgery treatment.

**Table 2:** Univariate analysis to identify independent variables that could affect the operation modality (*P* < 0.05 was identified with significant difference; n = number)

	Minimally invasive surgery n = 63 (10.86%)	Palliative surgery n = 460 (79.31%)	Radical surgery n = 57 (9.83%)	*P* value
**Gender**				
Male	36	258	36	
Female	27	202	21	*P* = 0.120
**Age (year)**				
≤44	6	59	3	
45–59	21	167	31	
60–74	32	203	23	
75–89	4	31	0	*P* = 0.059
**Spinal metastatic site**				
Cervical vertebra	0	42	5	
Thoracic vertebra	17	152	21	
Lumbar vertebra	16	122	8	
Sacral vertebra	3	28	4	
Trans‐segmental metastasis	27	116	19	*P* = 0.078
**Frankel score**				
A–C	3	75	12	
D	33	195	29	
E	27	190	16	*P* = 0.067
**KPS score**				
10–40	0	30	3	
50–70	21	209	34	
80–100	42	221	20	***P* = 0.017**
**SINS score**				
1–6	8	84	3	
7–12	42	327	24	
13–18	13	49	30	***P* < 0.001**
**VAS score**				
0–3	6	93	10	
4–6	26	223	36	
7–10	31	144	11	***P* = 0.009**
**Tokuhashi score**				
0–8	15	72	5	
9–11	31	221	20	
12–15	17	167	32	***P* = 0.021**
**Urinary and fecal incontinence**				
Yes	0	58	6	
No	63	402	51	***P* = 0.028**
**Primary tumor**				
Slow growth	16	113	12	
Moderate growth	23	182	18	
Rapid growth	24	165	27	*P* = 0.335
**Extraspinal metastasis**				
Yes	30	185	22	
No	33	275	35	*P* = 0.385
**Visceral metastasis**				
Yes	0	70	9	
No	54	390	48	*P* = 0.971
**Spinal pathological fracture**				
Yes	35	155	22	
No	28	305	35	***P* = 0.002**
**Bone lesion**				
Osteolytic	31	153	8	
Osteoblastic	0	8	1	
Mixed	1	2	0	
Unknown	31	297	48	***P* < 0.001**

### *KPS Score*

The KPS score was divided into three groups (10–40, 50–70, 80–100) (*P* = 0.017). For group 10–40, no patients received minimally invasive surgery, 30 (5.17%) patients received palliative surgery, and three (0.52%) patients received radical surgery. For group 50–70, 21 (3.62%) patients received minimally invasive surgery, 209 (36.03%) patients received palliative surgery, and 34 (5.86%) patients received radical surgery. For group 80–100, 42 (7.24%) patients received minimally invasive surgery, 221 (38.10%) patients received palliative surgery, and 20 (3.45%) patients received radical surgery.

### *SINS Score*

Three groups (1–6, 7–12, 13–18) (*P* < 0.001) were included for the SINS score. For group 1–6, eight (1.38%) patients received minimally invasive surgery, 84 (14.48%) patients received palliative surgery and three (0.52%) patients received radical surgery. For group 7–12, 42 (7.24%) patients received minimally invasive surgery, 327 (56.38%) patients received palliative surgery, and 24 (4.14%) patients received radical surgery. For group 13–18, 13 (2.24%) patients received minimally invasive surgery, 49 (8.45%) patients received palliative surgery, and 30 (5.17%) patients received radical surgery.

### *VAS Score*

The VAS score was divided into three groups (0–3, 4–6, 7–10) (*P* = 0.009). For group 0‐3, six (1.03%) patients received minimally invasive surgery, 93 (64.58%) patients received palliative surgery, and 10 (1.72%) patients received radical surgery. For group 7–12, 26 (4.48%) patients received minimally invasive surgery, 223 (38.45%) patients received palliative surgery, and 36 (6.21%) patients received radical surgery. For group 13–18, 31 (5.34%) patients received minimally invasive surgery, 144 (24.8%) patients received palliative surgery, and 11 (1.90%) patients received radical surgery.

### *Tokuhashi Score*

The Tokuhashi score was divided into three groups (0–8, 9–11, 12–15) (*P* = 0.021). For group 0–8, 15 (2.59%) patients received minimally invasive surgery, 72 (12.41%) patients received palliative surgery, and five (0.86%) patients received radical surgery. For group 9–11, 31 (5.34%) patients received minimally invasive surgery, 221 (38.10%) patients received palliative surgery, and 20 (3.45%) patients received radical surgery. For group 12–15, 17 (2.93%) patients received minimally invasive surgery, 167 (28.79%) patients received palliative surgery, and 32 (5.52%) patients received radical surgery.

### *Urinary and Fecal Incontinence*

Among these 580 patients, 64 (11.03%) patients presented urinary and fecal incontinence including 58 (10.00%) patients receiving palliative surgery and six (1.03%) patients receiving radical surgery. The remaining 516 (88.97%) patients were with no urinary and fecal incontinence, 63 (10.86%) patients received minimally invasive surgery, 402 (69.31%) patients received palliative surgery, and 51 (8.79%) patients received radical surgery. The difference was significant among groups (*P* = 0.028).

### *Spinal Pathological Fracture*

Two hundred and twelve (36.55%) patients presented spinal pathological fracture, and among these patients 35 (6.03%) patients received minimally invasive surgery, 155 (26.72%) patients received palliative surgery, and 22 (3.79%) patients received radical surgery. And while spinal pathological fracture did not occur in 368 (63.45%) patients, 28 (4.83%) patients received minimally invasive surgery, 305 (52.59%) patients received palliative surgery, and 35 (6.03%) patients received radical surgery. The difference was significant among groups (*P* = 0.002).

### *Bone Lesion (Osteolytic, Osteoblastic, or Mixed)*

Totally, 192 (33.10%) patients presented with osteolytic lesions through imaging examinations and received surgery treatment. Thirty‐one (5.34%) patients received minimally invasive surgery, 153 (26.38%) patients received palliative surgery, and eight (1.38%) patients received radical surgery. For patients with osteoblastic lesions, only eight (1.38%) patients received palliative surgery and one (0.17%) patient received radical surgery. For patients with mixed lesions, just one (0.17%) patient received minimally invasive surgery and two (0.34%) patients received palliative surgery. The difference was significant among groups (*P* < 0.001).

## Discussion

Spinal metastases are the most common type of bone metastasis with a prevalence of 30%–70% in cancer patients; 5%–10% of metastases may be associated with ESCC leading to impaired mobility, neurologic deficits, and decreased quality of life. However, there is still no consensus regarding the best treatment modality for these lesions. In this multicenter study, a total of 580 patients with an average age of 58.26 years (range, 13–86 years old) were enrolled in the study to summarize and analyze the epidemiological characteristics and independent variables affecting surgical modalities for spinal metastases.

Among these 580 patients, the epidemiological characteristics were analyzed. Three hundred and thirty two male and 248 female patients were enrolled with a ratio of 1.34:1, and most patients were at middle or elderly age between 45 years and 74 years. For primary lesion, the most common were lung cancer, followed by kidney cancer, breast cancer, gastrointestinal cancer, lymphoma and myeloma, prostate cancer, mesenchymal tissue sarcoma, and thyroid cancer. Especially, lung cancer was the top one leading to spinal metastasis either in males or females, which was different from data published abroad (prostate cancer in males and breast cancer in females). It may be due to the regional and cultural differences^23^. The most common spinal metastatic site was the thoracic vertebra (190 [32.76%]), followed by the lumbar vertebra (146 [25.17%]), and metastases involving more than two sites of the cervical, thoracic, lumbar, and sacral vertebrae arose in 162 (27.93%) patients, that was the same as in the report by Bollen *et al*.^24^.

As shown in Table 2, the KPS score, SINS score, VAS score, Tokuhashi score, urinary and fecal incontinence, spinal pathological fracture, and occurrence of bone lesion (osteolytic, osteoblastic or mixed) were independent and favorable factors affecting the surgery modalities. It could be determined that patients who received minimally invasive surgery preferentially should have a good general condition, the KPS score was more than 70 without urinary and fecal incontinence and visceral metastasis. Spinal metastatic sites showed no significant difference, but subgroup of vertebral body metastasis and appendix metastasis was not analyzed. However, some investigators pointed out that the minimally invasive surgery should be carefully selected for patients with vertebral body posterior wall and pedicle involvement^25^, so further analyses were needed to determine minimally invasive surgery indications for different spinal metastatic sites.

Unlike primary spinal tumors, the goal of surgery for spinal metastases is not cure but an overwhelming improvement of symptoms.^26^, ^27^, ^28^, ^29^ That is to say, surgeons must consider the patients’ overall health, as well as the imaging examination of the vertebral metastases. In this study, 460 (79.31%) patients received palliative surgery including 290 posterior laminectomy, 155 subtotal corpectomy, and 15 subtotal corpectomy combined with microwave ablation and vertebroplasty. Most of them presented severe pain and spinal instability but the general conditions were good with KPS score more than 60 and Frankel score in D and E. The revised Tokuhashi score have suggested that surgery only be considered in patients with a life expectancy of more than 6 months^30^, ^31^, meaning that patients, especially those with aggressive primary tumor metastasis, are ineligible for surgical symptom palliation^29^, ^32^. However, in this multicenter case series, lung cancer was the most common metastasis, as seen in 198 patients. Rapid development of radiotherapy and chemotherapy, especially targeted therapy, may help to improve patients’ life expectancy.

Radical surgery was also performed for spinal metastasis, but the complex anatomical structure of the spine made the operation very difficult and bleeding occurs frequently during the operation. Therefore, indications and contraindications should be strictly clear. The indications for spinal metastatic tumor resection are generally as follows: single‐level metastatic tumors of thoracic and lumbar vertebra with well‐controlled primary lesions susceptible to chemotherapy or targeted therapy; without vital visceral metastasis; patients with longer life expectancy; no more than two adjacent segment lesions; Tokuhashi score at a range of 12~15^22^, ^33^, ^34^. Only 57 (9.83%) patients who received tumor resection containing 36 total vertebrectomy and 21 total en‐bloc spondylectomy were enrolled in this retrospective study, most of them were met with the indications above. In addition, univariate analysis identified that patients with spinal pathological fracture and spinal instability (SINS score at a range of 13–18) could also be treated with tumor resection which should be considered for indications.

The limitations of this retrospective study include: lack of non‐surgical patients enrolled as control group; spinal metastatic sites are just on the basis of cervical vertebra, thoracic vertebra, lumbar vertebra, sacral vertebra, and trans‐segmental metastasis, however, another subgroup containing vertebral body and appendix should also be considered; and surgery modalities are not divided into the subgroup of operation combining with or without radiotherapy, chemotherapy and targeted therapy.

### *Conclusions*

Surgical treatment for spinal metastases is mainly to relieve pain, rebuild spinal stability, improve nerve function, control local tumors, and improve the quality of life of patients. With the rapid development of radiotherapy, chemotherapy (especially targeted therapy), immunotherapy and endocrine therapy, the level of surgical treatment of spinal metastases has been greatly improved. For middle‐aged and elderly patients with good general conditions, severe pain, spinal pathological fracture, spine instability and without urinary and fecal incontinence, early surgical treatment should be actively carried out.
